# Analysis of androgen receptor expression and activity in the mouse brain

**DOI:** 10.1038/s41598-024-61733-9

**Published:** 2024-05-15

**Authors:** D. Alwyn Dart, Charlotte L. Bevan, Pinar Uysal-Onganer, Wen Guo Jiang

**Affiliations:** 1 UCL (University College London) Cancer Institute, University College London, Paul O’Gorman Building, 72 Huntley Street, London, WC1E 6DD UK; 2 Department of Surgery and Cancer, Imperial Centre for Translational and Experimental Medicine, Imperial College London, London, W12 0NN UK; 3 Cancer Mechanisms and Biomarkers Research Group, School of Life Sciences, College of Liberal Arts and Sciences, University of Westminster, 115 New Cavendish Street, London, W1W 6UW UK; 4 Cardiff China Medical Research Collaborative, School of Medicine, Cardiff University, Cardiff, CF14 4YS UK

**Keywords:** Molecular neuroscience, Prostate cancer

## Abstract

Androgen deprivation therapy (ADT) is the core treatment for advanced prostate cancer (PCa), with a proven survival benefit. ADT lowers circulating testosterone levels throughout the body, but with it comes a variety of reported side effects including fatigue, muscle wastage, weight gain, hot flushes and importantly cognitive impairment, depression, and mood swings. Testosterone has a key role in brain masculinization, but its direct effects are relatively poorly understood, due both to the brain’s extreme complexity and the fact that some of testosterone activities are driven via local conversion to oestrogen, especially during embryonic development. The exact roles, function, and location of the androgen receptor (AR) in the adult male brain are still being discovered, and therefore the cognitive side effects of ADT may be unrecognized or under-reported. The age of onset of several neurological diseases overlap with PCa, therefore, there is a need to separate ADT side effects from such co-morbidities. Here we analysed the activity and expression level of the AR in the adult mouse brain, using an ARE-Luc reporter mouse and immunohistochemical staining for AR in all the key brain regions via coronal slices. We further analysed our data by comparing to the Allen Mouse Brain Atlas. AR-driven luciferase activity and distinct nuclear staining for AR were seen in several key brain areas including the thalamus, hypothalamus, olfactory bulb, cerebral cortex, Purkinje cells of the cerebellum and the hindbrain. We describe and discuss the potential role of AR in these areas, to inform and enable extrapolation to potential side effects of ADT in humans.

## Introduction

The foundations of prostate cancer (PCa) treatment are surgery, radiation, and androgen deprivation therapy (ADT). The global incidence of PCa is dramatically increasing, with over a million new cases each year^1^, suggesting that approximately 25 million men may be undergoing ADT at any moment. As androgens are active in many areas of the body, outside the reproductive system^2^, ADT is linked with substantial adverse effects, reducing the quality of life—including reduced muscle mass and bone mineral density, development of insulin resistance, increased fat mass, muscle atrophy, cardiovascular disease, sexual dysfunction, and cognitive impairment and decline^3^. Age of onset for several neurological diseases, *e.g.*, Alzheimer’s and dementia, coincide with PCa^4^, and distinguishing the ADT effects from pre-existing conditions is difficult, especially as the primary functions and effects of testosterone on the brain are complex and poorly understood. More generally, steroids can affect mood, emotional state, and brain function in humans, and in men with low testosterone, its replacement can greatly improve these symptoms^5^. Therefore, there is a pressing need to understand the brain regions that rely on testosterone for correct function and how ADT could disrupt this.

Brain development exhibits sexual dimorphism, as a result of gonadal steroids’ affects during specific perinatal stages^6^. In mammals, the default developmental pattern is female, and then testosterone derived from foetal testes, cause a permanent ‘male pattern’ brain differentiation and defeminisation^7^. However, a significant amount of male-specific brain differentiation is, in fact, mediated by the estrogen receptor (ER). Oestrogens (of which the major circulating form is oestradiol/E_2_) are locally synthesised in the foetal brain, from androgens, via aromatase enzyme^8^. Some brain regions show androgen receptor (AR) expression, and testosterone (T) is likely to have additional direct effects, independent of its conversion to E_2_^9^.

Early androgen exposure permanently changes brain physiology and circuitry, consequentially showing the sexually dimorphic ‘male-type’ behavioural pattern *e.g.,* sex, mating behaviour and aggression. Selected brain regions have been shown to require oestrogen to androgen conversion for full development *e.g.,* the sexually dimorphic nucleus of the preoptic area and the anteroventral periventricular nucleus^10^. However, aromatisation does not explain all the sexual differentiation in brain morphology. Human males with inactivating aromatase mutations show masculinised brains pattern, indicating a restricted ER function^11,12^. How much aromatase activity is limited to the embryonic, or adult, brain is unknown. Complete Androgen Insensitivity Syndrome (CAIS) is due to complete loss of AR function: CAIS individuals present with feminine appearance and brain pattern, despite functional ER and normal levels of testosterone, secreted by internalised testes^13^. Male mice with a brain-specific AR knock-out (hippocampus, medial amygdala, striaterminalis, preoptic area and the hypothalamus) displayed an effective absence of male-specific sexual behaviour even at relatively high levels of testosterone^7,14^.

The potential role of testosterone, versus its metabolites, on cognition remains controversial and requires further research. Men with moderate increases in testosterone displayed improvements in memory, whilst men with either large or small increases failed to do so^15,16^. Neurocognitive impairment and toxicity due to ADT is being acknowledged more in the clinic but are still poorly characterized with little information on their neurophysiological causes^17,18^.

The ADT treatments may also affect the brain differently. Androgen receptor antagonists (*e.g.,* bicalutamide) would not affect conversion of T to E_2_, whereas pituitary down regulators (GnRH agonists or antagonists) switch off T synthesis so would deprive the brain of both hormones. Some brain areas can express the biosynthetic enzymes required for steroid synthesis, making this even more complex, and may fall under the influence of steroid biosynthetic inhibitors used in ADT, such as abiraterone.

The use of an AR-driven luciferase reporter mouse model, with bioluminescence reflecting AR activity in all tissues, facilitates AR transcriptional activity analysis and differentiates the T > E_2_ conversion effects mediated by aromatase and ER. Previously, AR-driven bioluminescence was seen in several distinct regions of the mouse brain, with subtle differences between the sexes^19^. Here in this paper, in the hope of casting new light on the regional expression, and functionality of the AR in the mouse brain, we have coupled the AR-driven luciferase data with immunohistological staining for AR protein in sections covering the mouse brain in detail. Furthermore, we have correlated the results with the Allen Mouse Brain Atlas, which has AR mRNA expression ISH data, and a detailed map of brain morphological regions which will aid in mapping functional AR protein expression in fine detail.

## Materials and methods

### In vivo studies

Mouse tissue was obtained from adult male and female AR-Luc mice^19^ (background strain C57BL/6J) at 15–20 weeks old approx. Animals were free to receive food and water. Work was conducted under the provisions of the Animals (Scientific Procedures) Act 1986 of the United Kingdom (HMSO, London, UK, 1990) and was approved by Imperial College Animal Welfare and Ethical Review Body.

### Luciferase imaging

Mice were injected *i.p.* with *D*-luciferin (Caliper Life Sciences Ltd, Runcorn, UK) at 150 mg/kg, 10 min before imaging. After sacrifice, whole brain tissue was collected by surgical resection via the cranium. Tissue was rinsed in saline and imaged immediately. A mouse brain slicer matrix (Zivic Instruments, Pittsburgh, PA, USA) was used for coronal brain sections. The IVIS Imaging System 100 series (Perkin Elmer, MA, USA) was used for light detection and analysis. Images were collected as a greyscale image with light intensity overlaid as a pseudocolour image.

### Histology and immunohistochemistry

Post luciferase imaging, tissues were formaldehyde-fixed and embedded in paraffin wax blocks. Coronal sections (6 μm thickness) were sectioned. Sections were dewaxed and rehydrated and immersed for 15 min in H_2_O_2_ (1%), to quench endogenous peroxidase activity. Sections were microwaved for 5 × 5 min (750 W) in 10 mM citric acid buffer (pH 6.0), for nuclear antigen retrieval. Sections were washed and blocked in 10% goat serum (Vector, Peterborough, United Kingdom). Primary antibodies used were AR (N-20 Santa Cruz Biotechnology, TX, USA) @1:300. Sections were washed and incubated with 2^0^ goat anti-rabbit antibody (Agilent Technologies, CA, USA, @1/300). An avidin–biotin complex (Vector Labs, Peterborough, U.K.) was used for detection, using diaminobenzidine chromogenic substrate. The sections were counterstained with haematoxylin. Negative controls lacked primary antibody. Digital images were captured using E1000 microscope (Nikon, Kingston upon Thames, UK). Positive controls for AR were sections of mouse prostate tissue stained alongside. Sections were mapped to the Allen Mouse Brain Atlas (atlas.brain-map.org) for tissue structure, and to the AR ISH brain map for AR mRNA expression (http://mouse.brain-map.org/experiment/show/2039).

### Imaging and analysis

Images were analysed in ImageJ (version 1.54f, ImageJ: Rasband, W.S., ImageJ, U. S. National Institutes of Health, Bethesda, Maryland, USA, https://imagej.net/ij/, 1997–2018), using colour deconvolution (Hematoxylin & Dab) and image threshold adjustment, with conversion to binary image using the Fiji plug-in^20,21^.These AR staining density images are given in Supplemental Figs. 1–3. ImageJ was used to measure the number of AR positive nuclei in ten fields of view in the specific regions listed in Table 1, and expressed as a percentage of whole nuclei. Manual cell counting was also conducted.

**Table 1:** AR immunostaining location and intensity in different brain regions, of the male adult mouse.

Brain area	Sub area	AR staining type seen
Main olfactory bulb	Glomerular layer	Weak cytoplasmic staining, 100% of cells, occasional strong nuclear staining in 1% of cells
	Outer Plexiform layer	No detectable staining
	Mitral cell layer	Strong nuclear and cytoplasmic staining, in 95% cells
	Inner plexiform layer	Weak/occasional nuclear staining, 1–5% of cells
	Granule cell layer	Strong nuclear staining in occasional cells, ranging from 5% outer to 40% inner cells
	Olfactory limb	Strong nuclear staining in 70% of cells
Accessory olfactory bulb	Glomerular layer	Very weak cytoplasmic staining in all cells, no nuclear staining
	Mitral cell layer	Moderate nuclear staining in 50% cells
	Granule cell layer	Moderate nuclear staining in 50% cells
Cerebral cortex	Secondary motor area L1	No staining
	Secondary motor area L2/3	Strong nuclear staining—10% increasing to 80% of cells
	Secondary motor area L5	Strong nuclear staining, in 90% of cells
	Secondary motor area L6	Strong nuclear staining in 90% of cells
	Primary motor area L1	No staining
	Primary motor area L2/3	Strong nuclear staining in 50–90% of cells
	Primary motor area L5	Strong nuclear staining in 90% of cells
	Primary motor area L6	Strong nuclear staining in 90% of cells
	Primary somatosensory area L1	No staining
	Primary somatosensory area L2/3	Strong nuclear staining in 50–90% of cells
	Primary somatosensory area L4	Strong nuclear staining in 50–90% of cells
	Primary somatosensory area L5	Strong nuclear staining in 70–90% of cells
	Primary somatosensory area L6	Strong nuclear staining in 70–90% of cells
	Gustatory area L1	No staining
	Gustatory area L2/3	Strong nuclear staining in 50–90% of cells
	Gustatory area L4	Strong nuclear staining in 50–90% of cells
	Gustatory area L5	Strong nuclear staining in 70–90% of cells
	Gustatory area L6	Strong nuclear staining in 70–90% of cells
	Ventral auditory & temporal area L1	No staining
	Ventral auditory & temporal area L2/3	Strong nuclear staining in 25–60% of cells
	Ventral auditory & temporal area L4	Strong nuclear staining in 25–60% of cells
	Ventral auditory & temporal area L5	Strong nuclear staining in 25–60% of cells
	Ventral auditory & temporal area L6	Strong nuclear staining in 25–60% of cells
	Ectorhinal, perirhinal & entorhinal areas L1	No staining
	Ectorhinal, perirhinal & entorhinal areas L2/3	Strong nuclear staining in 25% of cells
	Ectorhinal, perirhinal & entorhinal areas L4	Strong nuclear staining in 25% of cells
	Ectorhinal, perirhinal & entorhinal areas L5	Strong nuclear staining in 25% of cells
	Ectorhinal, perirhinal & entorhinal areas L6	Strong nuclear staining in 25% of cells
	Piriform layers 1–3	No staining in outer layer, strong nuclear stain in 50–70% of cells in inner layers
Amygdala	Cortical, piriform and medial amygdala areas	No staining (cortical), moderate nuclear staining in 20% piriform layer, strong nuclear staining in 70% of cells (medial)
Basal ganglia	Caudoputamen	Weak to moderate nuclear staining in 20–50% of cells
	Nucleus accumbens	Weak nuclear staining in occasional cells 5–10%
	Olfactory Tubercle	Moderate nuclear staining in 70% of cells
Hippocampus	Stratum oriens	Weak nuclear staining in 20% of cells
	Pyramidal layer	Moderate cytoplasmic and nuclear staining in occasional cells
	Molecular layer	Occasional nuclear staining 5% cells
	Granule cell layer	Single layer of nuclear staining cells, no staining in surrounding cells,
Thalamus	Lateral, dorsal, ventral, & posterior area	Nuclear staining in 25% of all cells
	Medial area	Nuclear staining in 40% of all cells
Hypothalamus	Lateral area	Nuclear staining in 50% of all cells
	Dorsal area	Nuclear staining in 50% of all cells
	Ventromedial	Nuclear staining in 50% of all cells
	Arcuate	Moderate nuclear staining in 70% of all cells
Midbrain	Sensory related	Weak to moderate nuclear staining in 10% of cells
	Motor related	Weak to moderate nuclear staining in 10% of cells
Hindbrain	Pons & fibre tracts	Moderate nuclear staining in 70% of cells
Cerebellum	Molecular layer	No staining
	Purkinje layer	Strong nuclear specific staining in all cells
	Granular layer	Weak cytoplasmic staining in 40% of cells
Medulla	Parvicellular Reticular nucleus	Moderate to strong cytoplasmic and nuclear staining in 5–10% of cells
	Spinocerebral tract	Moderate to strong cytoplasmic and nuclear staining in 20% of cells
	Medial longitudinal fascicle	Moderate to strong cytoplasmic and nuclear staining in 30% of cells

### Ethics statement

All mouse procedures were performed in accordance with the UK Animals (Scientific Procedures) Act 1986 under Home Office license.

### Ethics approval and consent to participate

Work was conducted under the provisions of the Animals (Scientific Procedures) Act 1986 of the United Kingdom (HMSO, London, UK, 1990) and was approved by Imperial College Animal Welfare and Ethical Review Body. All methods were carried out in accordance with relevant guidelines and regulations and within ARRIVE guidelines.

## Results and discussion

### AR Activity in the adult male and female mouse brain

**Figure 1:**
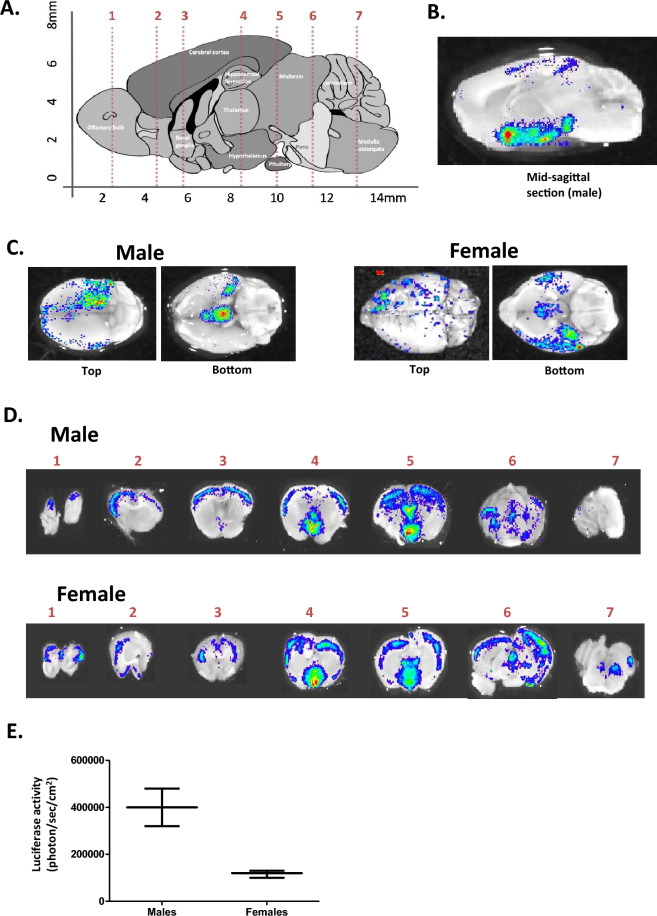
Androgen receptor transcriptional activity in male and female brain tissue as monitored by luciferase reporter activity. (**A**) Schematic sagittal diagram of the main structures in the mouse brain. Approximate average size is given on the axis. Numbered red lines represent the main coronal sections presented in later figures. (**B**–**D**) Bioluminescent imaging of male and female ARE-Luc mice, injected with 150 mg/kg luciferin substrate, and imaged with a CCCD camera after 10 min. Image represents a greyscale photograph overlaid with a pseudocolour representation of bioluminescence. (**B**) Mid-sagittal section of male mouse brain. (**C**) Whole mouse brain tissues from male (left hand side) and female (right hand side), showing light emission looking down at the top, and up from the bottom. (**D**) Coronal sections of male (upper panel) and female (lower panel) brain tissues, representing those areas highlighted in 1A (coronal sections 1–7). (**E**) Graph indicating the average measured bioluminescent activity per cm^2^ from male and female mouse brain tissues sections, error bars represent data from three mice in each group.

In the ARE-Luc mouse, AR transcriptional activity drives expression of a stably integrated luciferase gene, via a synthetic AR-responsive promoter. Hence AR activity, in all AR-expressing tissues, can be measured as bioluminescence from live animals or ex vivo tissues. Figure 1A, shows the layout of the mouse brain, with the main coronal example sections presented in this paper (1–7, from the olfactory bulb to the medulla), in which we examined in detail the AR staining and activity.

Whole brain tissues or coronal brain slices were imaged for luciferase activity. Figure 1B shows the whole brain in central sagittal section, showing strong bioluminescence from the hypothalamic and pituitary regions. Figure 1C shows whole brain tissues from male and female mice, visualised from above and below. Figure 1D, shows progressive coronal sections 1 through 7 from male and female mice, demonstrating distinct luciferase activity in neuronal layers and regions within the brain of both sexes. Luciferase activity was seen in the olfactory bulb and cerebral cortex, within distinct neuronal cell layers. Luciferase activity was very strong in the hypothalamus, thalamus, and pituitary gland (Fig. 1D), and relatively very low in the cerebellum. As expected, luciferase levels were generally lower (approximately four-fold) in the female mouse brain (Fig. 1E), being especially lower in the thalamus and hypothalamus regions (see Fig. 1D).

### Histological staining for AR protein expression in brain coronal sections

**Figure 2:**
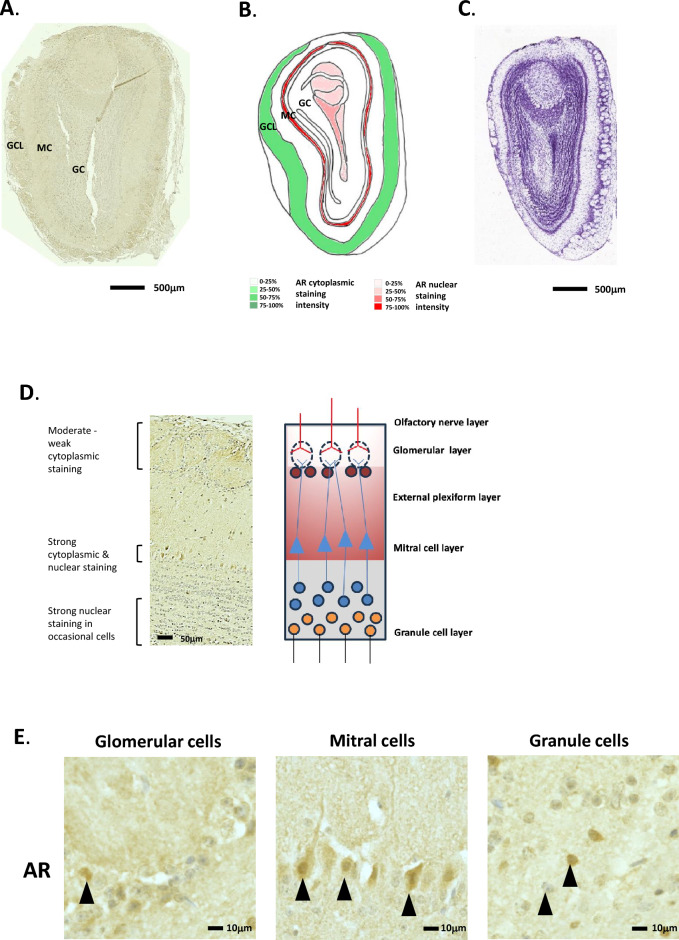
Androgen receptor expression in the mouse olfactory bulb. (**A**) Immunohistochemical staining for AR in the mouse olfactory bulb, whole coronal section shown. (**B**) Schematic diagram of the mouse olfactory bulb, as modified from the Allen Brain Atlas for regional reference. Red colour represents a summary of the AR staining seen and level of intensity. (**C**) Haematoxylin and eosin-stained section of the mouse brain as taken from the Allen Brain Atlas (Coronal image 20). (**D**) Immunohistochemical staining for AR in a cross-sectional view of the cellular layers of the olfactory bulb. Associated schematic image (right hand side) given for reference. (**E**) High magnification images of the different cell types in the olfactory bulb showing immunohistochemical staining for AR expression. GCL, glomerular cell layer; MC, mitral cell layer; GC, granule cell layer.

**Figure 3:**
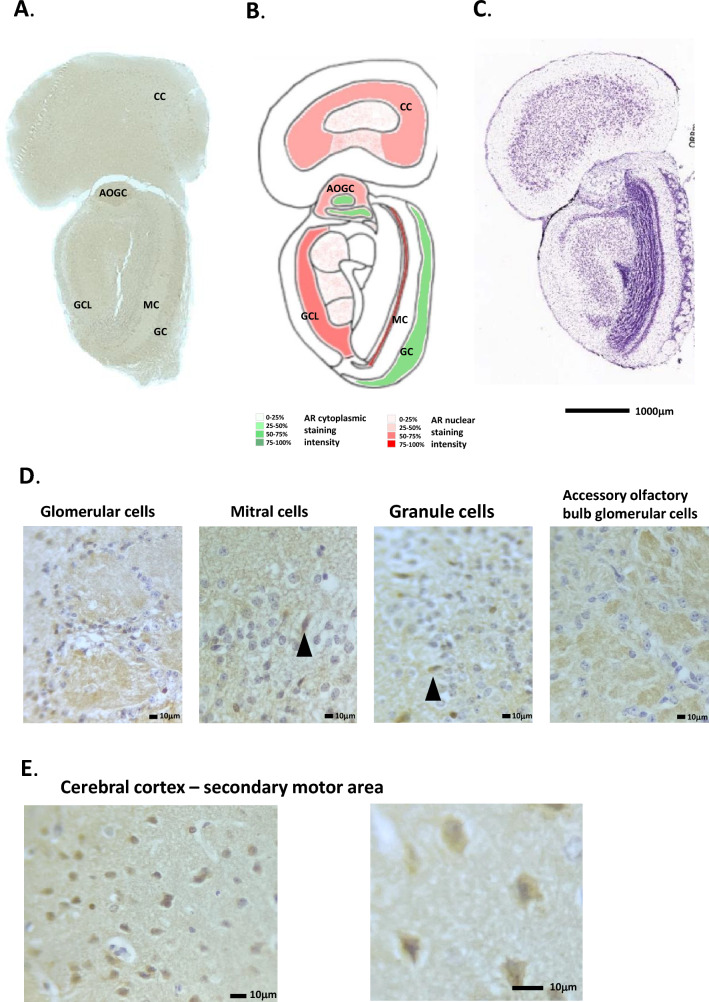
Androgen receptor expression in the mouse olfactory bulb, accessory olfactory bulb and cerebral cortex. (**A**) Immunohistochemical staining for AR in the mouse olfactory & accessory olfactory bulbs, whole coronal section shown. (**B**) Schematic diagram of the mouse olfactory bulb & accessory olfactory, as modified from the Allen Brain Atlas for regional reference. Red colour represents a summary of the AR staining seen and level of intensity. (**C**) Haematoxylin and eosin-stained section of the mouse brain as taken from the Allen Brain Atlas (Coronal image 24). (**D**) High magnification images of the different cell types showing immunohistochemical staining for AR expression. (**E**) High magnification images of the mouse frontal cerebral cortex showing strong AR expression in the secondary motor area. GCL, glomerular cell layer; MC, mitral cell layer; GC, granule cell layer; AOGC, accessory olfactory bulb glomerular cell layer; CC, cerebral cortex.

**Figure 4:**
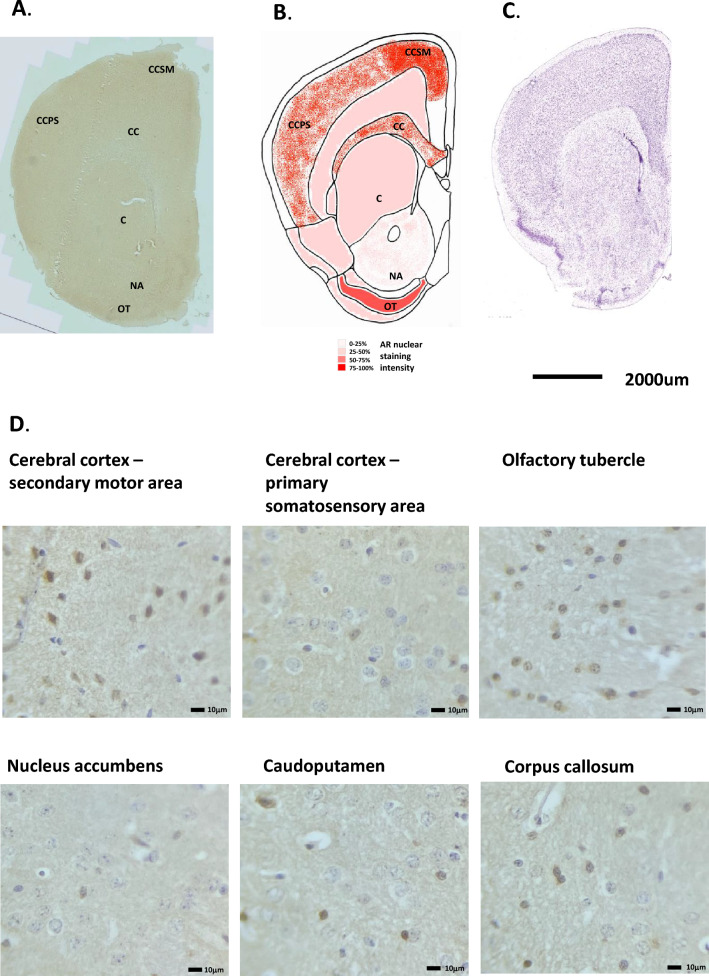
Androgen receptor expression in the mouse cerebral cortex and basal ganglia. (**A**) Immunohistochemical staining for AR in the mouse cerebral cortex and basal ganglia, whole coronal section shown. (**B**) Schematic diagram of the mouse cerebral cortex and basal ganglia, as modified from the Allen Brain Atlas for regional reference. Red colour represents a summary of the AR staining seen and level of intensity. (**C**) Haematoxylin and eosin-stained section of the mouse brain as taken from the Allen Brain Atlas (Coronal image 40). (**D**) High magnification images of the different cell types showing immunohistochemical staining for AR expression. CCSM, cerebral cortex secondary motor area; CCPS, cerebral cortex primary somatosensory area; CC, Corpus callosum; C, caudoputamen; NA, nucleus accumbens.

**Figure 5:**
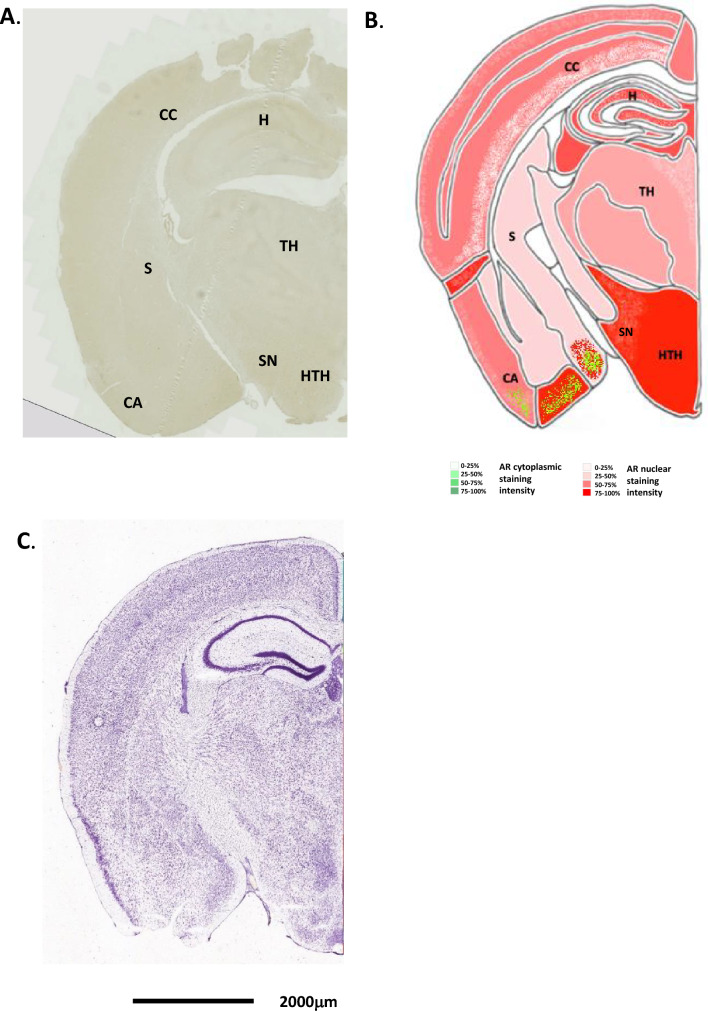
Androgen receptor expression in the mouse cerebral cortex, hippocampus, thalamus and hypothalamus. (**A**) Immunohistochemical staining for AR in the mouse cerebral cortex, hippocampus, thalamus and hypothalamus, whole coronal section shown. (**B**) Schematic diagram of the mouse cerebral cortex, hippocampus, thalamus and hypothalamus, as modified from the Allen Brain Atlas for regional reference. Red colour represents a summary of the AR staining seen and level of intensity. (**C**) Haematoxylin and eosin-stained section of the mouse brain as taken from the Allen Brain Atlas (Coronal image 71). CC, cerebral cortex; TH, thalamus; HTH, hypothalamus; S, striatum; CA, cortical amygdalar area.

**Figure 6:**
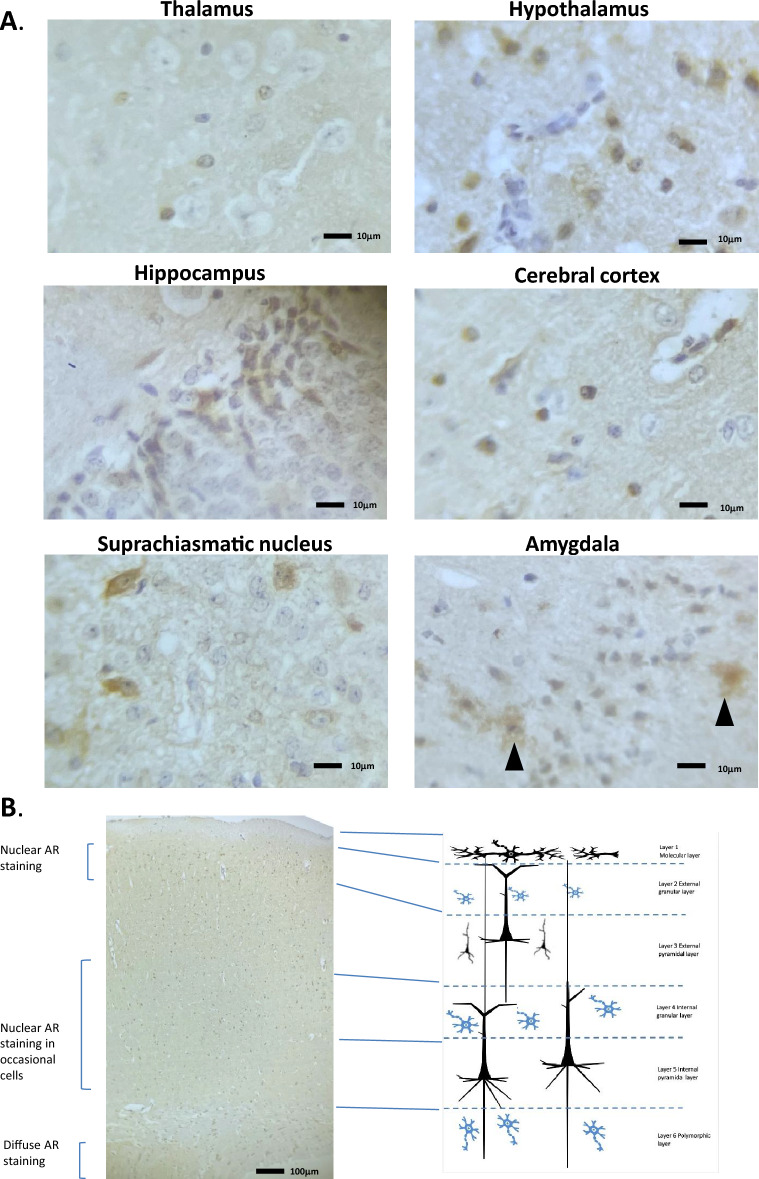
Androgen receptor expression in the thalamus, hypothalamus, hippocampus and cerebral cortex. (**A**) High magnification images of the different cell types showing immunohistochemical staining for AR expression. (**B**) Immunohistochemical staining for AR in a cross-sectional view of the cellular layers of the cerebral cortex. Associated schematic image (right hand side) given for reference.

**Figure 7:**
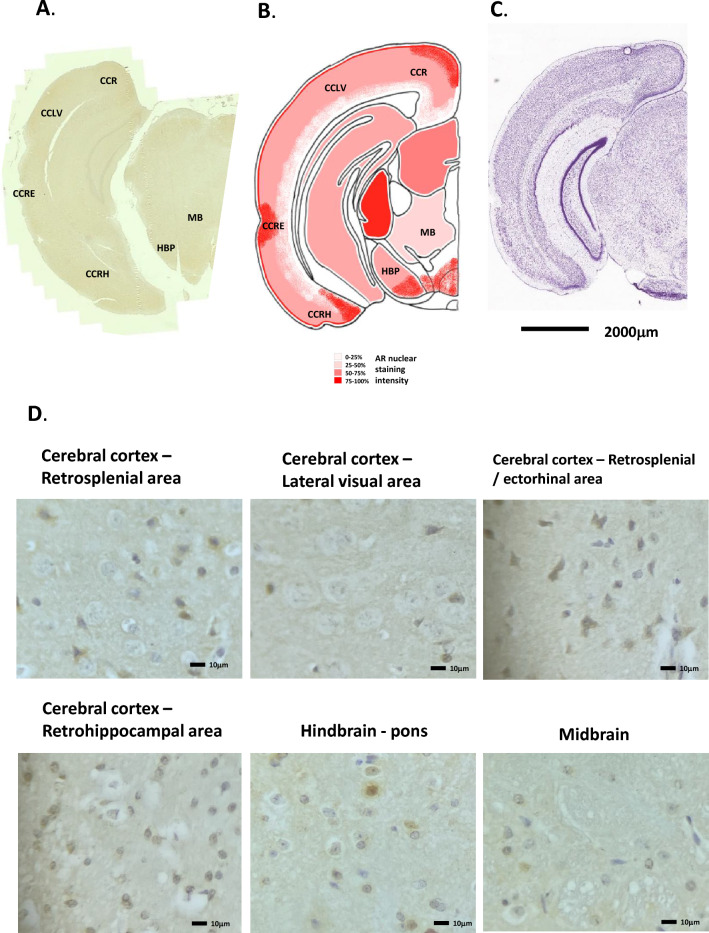
Androgen receptor expression in the mouse cerebral cortex, retrohippocampus, midbrain and hindbrain regions. (**A**) Immunohistochemical staining for AR in the mouse cerebral cortex, retrohippocampus, midbrain and hindbrain regions, whole coronal section shown. (**B**) Schematic diagram of the mouse cerebral cortex, retrohippocampus, midbrain and hindbrain regions, as modified from the Allen Brain Atlas for regional reference. Red colour represents a summary of the AR staining seen and level of intensity. (**C**) Haematoxylin and eosin-stained section of the mouse brain as taken from the Allen Brain Atlas (Coronal image 88). (**D**) High magnification images of the different cell types showing immunohistochemical staining for AR expression. CCR, cerebral cortex retrosplenial area; CCLV, cerebral cortex lateral visual area; CCRE, cerebral cortex retrosplenial/ectorhinal area; MB, midbrain; HBP, Hindbrain & Pons; CCRH, cerebral cortex retrohippocampal area.

**Figure 8:**
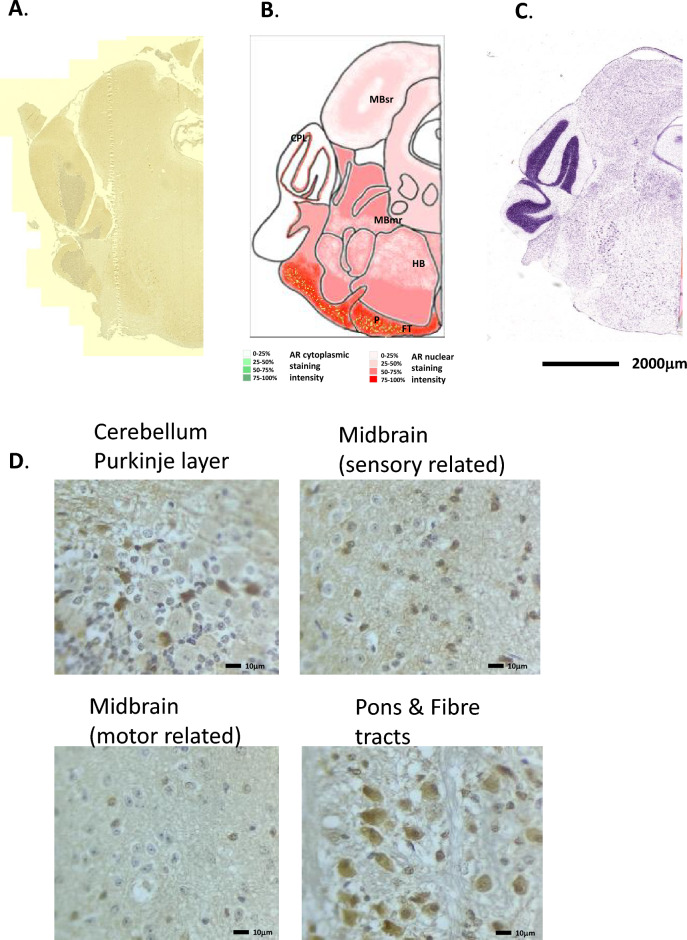
Androgen receptor expression in the mouse midbrain and hindbrain (Pons) regions. (**A**) Immunohistochemical staining for AR in the mouse midbrain and hindbrain regions, whole coronal section shown. (**B**) Schematic diagram of the mouse midbrain and hindbrain regions, as modified from the Allen Brain Atlas for regional reference. Red colour represents a summary of the AR staining seen and level of intensity. (**C**) Haematoxylin and eosin-stained section of the mouse brain as taken from the Allen Brain Atlas (Coronal image 105). (**D**) High magnification images of the different cell types showing immunohistochemical staining for AR expression. MBsr, Midbrain sensory related; MBmr, Midbrain motor related; CPL, cerebellum Purkinje layer; HB, hindbrain; P, Pons; FT, fibre tracts.

**Figure 9:**
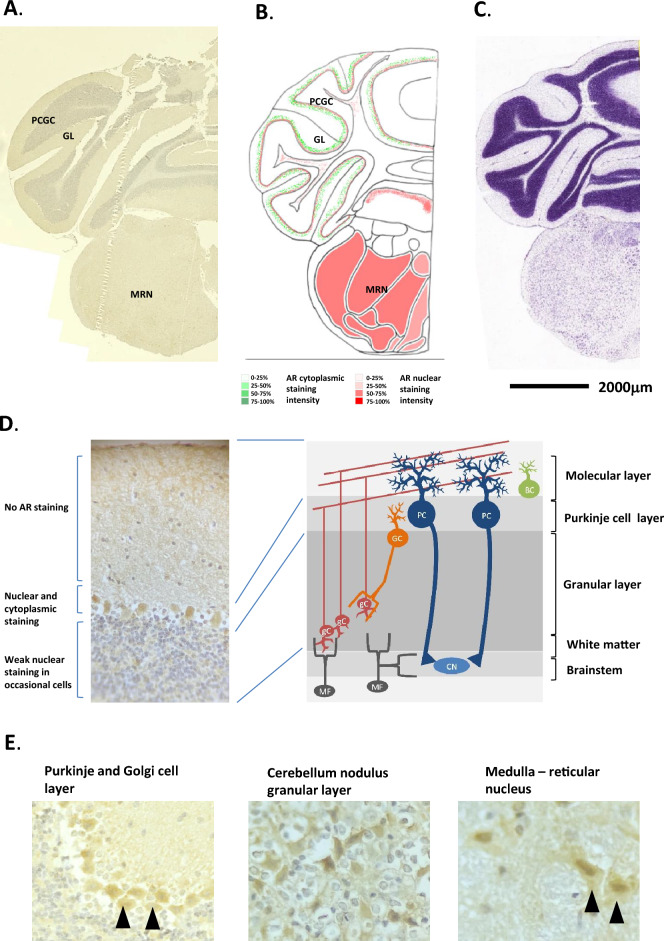
Androgen receptor expression in the mouse cerebellum and medulla. (**A**) Immunohistochemical staining for AR in the mouse cerebellum and medulla, whole coronal section shown. (**B**) Schematic diagram of the mouse cerebellum and medulla, as modified from the Allen Brain Atlas for regional reference. Red colour represents a summary of the AR staining seen and level of intensity. (**C**) Haematoxylin and eosin-stained section of the mouse brain as taken from the Allen Brain Atlas (Coronal image 127). (**D**) High magnification images of the different cell types showing immunohistochemical staining for AR expression, associated schematic image (right hand side) given for reference. (**E**) High magnification images of the different cell types showing immunohistochemical staining for AR expression. PCGC, Purkinje cell and Golgi cell layer; GL, granular layer; MRN, medullary reticular nucleus.

AR staining and density was analysed using Image J colour deconvolution of microscopy images. This was first tested using mouse normal prostate sections (Supplemental Fig. 1), and validated using no antibody controls for immunohistochemical staining for AR (Supplemental Fig. 2). The number of cells exhibiting cytoplasmic staining are given in green and the number of cells with nuclear specific AR staining are given in red, on Figs. 2, 3, 4, 5, 6, 7, 8 and 9. The results for each region from both Image J analysis and manual counting have been summarised in Table 1, and density images are seen in Supplemental Fig. 3A–G. Where appropriate, this indicates whether AR staining is nuclear or cytoplasmic. As AR is a transcription factor, nuclear AR would be expected in tissues where it is active.

### Olfactory bulb & accessory olfactory bulb

In the male mouse olfactory bulb (OB), strong and varied AR staining was seen in several tissue regions (example in Fig. 2A and summarised in B). H&E staining is given in Fig. 2C for reference (Fig. 2A–C). AR staining was seen to be weak to moderate in the glomeruli cells, which showed mainly cytoplasmic staining (Fig. 2D and E). In situ hybridisation of AR mRNA expression in the mouse olfactory bulb showed a similar pattern of expression (Allen Brain Atlas, ISH Coronal image 4). The mitral cells showed moderate to strong AR staining, in nuclear and cytoplasmic compartments (Fig. 2E). Weak to moderate nuclear staining was seen in the granule cell layer, which became more prevalent towards the centre of the OB (Fig. 2D and E). No staining was seen in the external plexiform layer. The OB tissue was too small to align luciferase activity regions directly.

In the next coronal section (section “Materials and methods”), the accessory olfactory bulb (AOB) can be seen, as well as the posterior part of the main OB (Fig. 3A, B). The AOB is structurally similar to the main OB, albeit smaller. H&E staining is given in Fig. 3C for reference. In situ hybridisation of AR mRNA expression in the mouse accessory olfactory bulb showed a similar pattern of expression (Allen Brain Atlas, ISH Coronal image 7). Here, AR cytoplasmic staining was seen in the glomerular cells, similar to that for the main OB, however, no distinct mitral cell staining could be seen, and the granule cell layer contained only occasional cells with nuclear AR staining (Fig. 3A, B, D and E). The overall AR staining in the posterior part of the main OB was also weaker than that seen in the anterior.

In the mouse, the olfactory system is composed of the OB and the peripheral olfactory system, with several separate anatomical sites for olfactory detection, each with distinct roles. The main olfactory epithelium which detects a variety of odorants, similar to humans, the vomeronasal organ (vestigial in humans) which detects non-volatile odorants (sex pheromones), the Grueneberg ganglion for alarm pheromones detection as well as predators’ scents, and the septal organ of Masara, whose function has been linked to odorants in social cues and mating (reviewed in^17^).

Peripheral olfactory neurones, in the nasal cavity, transmit their information directly to the OB, ending in glomerular clusters. Next is the external plexiform layer, which is made of dendrites of the glomeruli neurones and astrocytes. Lastly are the mitral cells which output the OB and link it to the olfactory cortex, which are modulated by the granular neuron layer^17^. However, other neural pathways exist e.g., the vomeronasal neurons may contact to the AOB, which are connected to the amygdala and the stria terminalis bed nucleus, which in turn connect to the anterior hypothalamus.

Androgens, via the AR, influence the development and regulation of synaptic plasticity, neuronal connectivity, dendrite pruning, synapse formation and the remodelling of neural circuits, thereby shaping the circuitry and function in the olfactory system^18^. AR can influence the sensitivity and response properties of olfactory sensory neurons, potentially affecting an animal's ability to detect and discriminate odours, and testosterone levels regulate AOB neurogenesis and activate the vomeronasal system, influencing sexual attraction and mating in mice^19^. Rodents depend greatly on olfactory signals for mating and reproductive behaviour, therefore, these neural circuits are clear targets for sexual differentiation.

In mice, chemical signatures are identified by two discrete pathways. The olfactory epithelium projects directly into the main OB, and the Vomeronasal Organ (VNO) projects directly to the AOB. It was, therefore, surprising to find that only weak (cytoplasmic) and minimal AR staining could be seen in the AOB, and that no detectable luciferase activity could be seen in the AOB area (Fig. 1C), in contrast to the relatively robust AR staining and activity seen in the main OB. Sex steroids change the sensitivity of VNO neurons to significant odourants e.g. pheromones that offer cues to the social and reproductive status of other animals. Sexually dimorphism in perception may start at the sensory receptors (VNO), which may not need to distinguish between different sensory inputs^20^, whereas the main OB, may require more complex neural processing as driven by sex hormones and their receptor activities. Research suggests that sex discrimination of urinary volatiles is mediated by the main OB, whereas its reward / behavioural response may be mediated by the AOB^21^. The Allen Mouse Atlas did not show strong expression of ERα & ERβ via ISH, suggesting that ER-masculinisation may be restricted to the embryonic phase but less relevant for the adult male.

The olfactory system in the human is not as highly developed as in mice or other mammals, and there is limited evidence that pheromones play any part in human mating behaviour, however, the olfactory system is regularly designated as the most "primitive" sensory system due to the direct connections to more primitive, subconscious regions of the brain (olfactory cortex and the limbic system), which are important in our emotional states, mood and in memory formation. These pathways could be targets for disruption by ADT.

### Cerebral cortex and basal ganglia

The mouse cerebral cortex can be seen across Figs. 3, 4, 5, 6 and 7 in the presented data. The cerebral cortex is organized into six main stacked layers (L1–L6), see Fig. 6B, (reviewed in^22^).

In the anterior portion of the cerebral cortex, (Fig. 3A, B and E), nuclear specific AR staining was observed in the inner layers (L2-3) of the orbital area (medial part), pre-limbic area, primary and secondary motor area and agranular insular area layers. Staining was seen in 80% of the cells approx. and was primarily nuclear with some degree of cytoplasmic staining. A much lower amount of positively staining nuclei were seen in the orbital area (lateral & ventrolateral parts). Figure 3E shows a high magnification image of the anterior cortex.

Moving through the cerebral cortex to the central areas above the basal ganglia, the pattern of AR staining is similar. Cells in the secondary motor area showed strong nuclear AR staining (Fig. 4A, B and D). In situ hybridisation of AR mRNA expression in the mouse accessory olfactory bulb showed a parallel expression pattern (Allen Brain Atlas, ISH Coronal image 15). H&E staining is given in Fig. 4C for reference. The remaining cerebral areas, including the primary somatosensory area, gustatory area, and agranular insular areas also showed numerous AR positive nuclei with varying degrees of staining. All staining was more prominent in layers L2-3.

The middle of the cerebral cortex, situated above the thalamus, and the posterior region of the cerebral cortex, again showed strong AR staining. The regions of the retrosplenial area (lateral & dorsal part), primary and secondary motor area, primary somatosensory areas, primary, ventral, and dorsal auditory area, temporal association areas all showed nuclear specific staining for AR in levels L2-4, with little or no staining in the lower layers L5-6 (Fig. 5A, B, 6A, B and 7A, B). This agreed well with the AR-driven luciferase signal seen in Fig. 1D. Strong nuclear staining was seen in the ectorhinal area, perirhinal area, entorhinal area, cortical amygdalar area, retrohippocampal area, and piriform amygdalae areas (Figs. 5A, B and 7A, B, D).

The cerebral cortex is a greatly folded neuronal tissue sheet that surrounds the cerebrum. It is vital for higher brain functionality e.g. cognition, memory, perception, consciousness and specifically for humans—speech and self-awareness. It processes sensory and motor inputs and communicates with other regions of the brain.

These layers are not strictly distinct in terms of their functions, as there is considerable interaction and overlap. Information arriving at the cortex is processed by a local circuit that crosses the layers (see Fig. 6B). The AR expression and activity was limited to the internal layers L2-5 primarily, in most areas, which may not be surprising as the outer layers are mainly dendritic bodies and not where we see the neuronal cell nucleus, where AR is presumably active.

The entorhinal cortex, located in the medial temporal lobe, has roles including a hub network for memory, spatial awareness, and time perception. Studies in rodents indicate this region’s importance for visible cues to conduct spatial awareness tasks. The retrosplenial cortex is exceptionally receptive to environmental markers, and for spatial judgements. The entorhinal cortex is the primary brain region to be affected in Alzheimer's disease^21^. In humans, studies show an expansive range of cognitive functions for the retrosplenial cortex including recall, navigation, and event/scene prediction. The retrosplenial centre has been closely linked with episodic memory loss and spatial awareness dysfunction in the early stages of Alzheimer’s before the onset of amyloid deposition^22^. The retrohippocampal region has been implicated in response to social recognition memory which, in rodents, may be driven primarily by odorant detection^23^.

The cortical amygdala controls innate odour-driven behaviours, and allows participation in learned, and adaptive olfactory behaviours. Links from the OB to cortical amygdala may drive innate odour-driven behaviours, whereas indirect links may drive learned odour responses^24^. The amygdala functions proper will be discussed later.

### Olfactory tubercle

The olfactory tubercle (OT) stained strongly for AR which was primarily nuclear (Fig. 4A, B and D). AR-driven luciferase activity was detectable in this area but was quite weak. (Fig. 1D).

As the OT is linked to the OB, stimulation by specific odorants can activate the OT to trigger motivation-driven behaviours, via accessing the limbic centres of the brain^21,22^. Specific tufted cell neurones in the OB connect directly to the OT^23^. Additionally, the OT receives inputs from the ventral tegmental area (VTA), indicating that the OT integrates these inputs to respond to olfactory stimuli based on previous experience.

In humans, the sense of smell can be strongly associated with emotional memories^24^, and associations to scents and aromas can contribute positively to good psychological health^25^, signifying a specialized form of olfactory-based memory in the limbic regions. As the OB and the OT are so closely linked, they form an ‘olfactory cortex’, where the OT functions in the integration and conversion of sensory signals into motivated behaviours. The OT is important in sexual responsiveness^26^ and may be critical site for social odour processing. Bilateral OT lesions significantly reduced copulatory behaviour in male rats and a reduced sniffing and chewing behaviours. Presence of both ER and AR further support this predicted function^27^ adjusting sensory olfactory processing in accordance with fluctuating sex hormone levels. Again, such highly AR-regulated neural pathways may be targets for ADT, which are important for our emotional states, mood and in memory formation.

### Basal ganglia (nucleus accumbens, caudoputamen, corpus callosum)

In the *nucleus accumbens* area of the mouse brain only weak, and occasional cell staining was seen for AR (Fig. 4A, B and D). Additionally, little luciferase activity could be seen in this area in fresh brain coronal sections (Fig. 1D). The extent of AR staining in the caudoputamen was intermittent. Some cells had moderate to strong nuclear specific staining whilst other cells did not stain for AR, see Fig. 4A, B and D. Luciferase activity was also weak in this area, (see Fig. 1D). The extent of AR staining in the corpus callosum was very intermittent. Some cells had moderate to strong nuclear specific staining whilst other cells did not stain for AR, see Fig. 4A, B and D. Luciferase activity was also weak in this area, (see Fig. 1D). H&E staining is given in Fig. 4C for reference.

The nucleus accumbens is critical in the brain's reward circuitry with functions related to motivation, pleasure, and reward stimuli in response to food, sex, and chemicals/drugs. The associated dopamine release creates pleasurable feelings and reinforces reward behaviours promoting their repetition. The nucleus accumbens may be involved in social reward processing, which can promote social bonding and cooperation.

Gonadal hormones can control and manipulate rewarding behaviour, especially when linked to mating e.g. copulation, aggression, and physical activity. Furthermore, they can affect susceptibility to addiction-like behaviours^21^. Gonadal hormones can modulate neuronal plasticity^22^ e.g. spine density, morphology and dendrite length, leading to sexual dimorphic development, where mating behaviour is naturally occurring, and dependent upon gonadal hormones. Gonadectomy decreases spine density and can be rescued by DHT treatment^22^.

The nucleus accumbens directly regulates male mouse behavioural responses to chronic social defeat stress, e.g., from contact with a more aggressive or powerful male mouse^23^. Additionally, testosterone levels can drop in response to stress of various types, and testosterone may influence risk taking and decision-making behaviour by altering dopamine function in nuclear accumbens^24^.

Although depressive disorders are more prevalent in females than males, depression increases in older males as plasma testosterone drops^25^. Alterations in nucleus accumbens, a region critical for reward and motivation, are implicated in the pathophysiology of depression^26,27^ and may be strongly affected by ADT.

The caudate nucleus and the putamen together form a structure known as the striatum, which is a key component of the basal ganglia. They have interconnected functions in motor control, coordination, cognitive functions, reward and reinforcement learning, emotion regulation and psychiatric disorders.

In healthy humans, dopamine release in the striatum differs between the sexes^21^, and testosterone administration has been linked to increased levels of dopamine^22^. In adolescents with male-dominant neurodevelopmental disorders, striatal dysfunction has been noted in attention deficit hyperactivity disorder (ADHD), pervasive developmental disorder and Tourette’s syndrome. Normal dopamine release and neural function may be strongly affected by ADT, leading to depression, and neurological suppression in cognition, coordination, and learning.

The corpus callosum links both brain hemispheres, allowing information transfer and functions in coordination and complex problem-solving. Corpus callosum agenesis can lead to a wide range of developmental disorders including physical e.g., vision impairments, seizures, delayed childhood development, and cognitive disorders. In rodents, the male corpus callosum is significantly larger than the female's, a dimorphism depending on the prenatal exposure to testosterone^21^. In humans, the corpus callosum increases in size and complexity during the pubertal androgen surge in males^22^. ADT has been shown to affect white matter integrity, associated with slower cognitive processing speed, supporting the deleterious effects of ADT on the brain and cognition in PCa patients^23^.

### Thalamus

In the male mouse the thalamus region showed a strong AR-driven luciferase activity (Fig. 1D). In the female mouse the signal was less strong and showed a slightly different physiological location for the activity. However, in the male mouse, although AR activity was strong, the expression was seen to be much weaker than seen in the hypothalamus, and only 40–50% approx. of the cells were AR-positive (Figs. 5B and 6A). In situ hybridisation of AR mRNA expression in the mouse accessory olfactory bulb showed a similar pattern of expression (Allen Brain Atlas, ISH Coronal image 29). H&E staining is given in Fig. 5C for reference.

The thalamus is located deep within the brain and has many important functions in the central nervous system. It acts as a relay station for sensory information, and processing and integrating sensory inputs, and contributes to the functions of the motor cortex, sleep regulation, emotion, and memory. The thalamus, along with other brain regions coordinate responses to fear, aggression, sexual and emotional responses in the male (reviewed in^21^). The high AR-driven luciferase activity seen did not correlate with the modest levels of AR expression observed.

### Hypothalamus

AR staining in the hypothalamus was strong, with around 90% of all cells staining positive, with primarily a nuclear specific pattern (Fig. 5A, B and 6A). AR-driven luciferase activity in this area was by far the highest seen for the mouse brain (Fig. 1D). H&E staining is given in Fig. 5C for reference.

The high expression and activity of AR in the hypothalamus is not surprising given it sits at the apex of steroid hormone production within the hypothalamic–pituitary–gonadal axis. Gonadotropin-releasing hormone (GnRH) is secreted from the hypothalamus, by GnRH-expressing neurons, into the artery supplying the pituitary gland. These are detected by the anterior pituitary which produces luteinizing hormone (LH) and follicle-stimulating hormone (FSH) into circulation which stimulates the testes to produce testosterone. Testosterone secreted by the testes (along with the hormone inhibin) acts as a negative feedback mechanism to maintain homeostasis.

However, although testosterone does indeed modulate GnRH release by controlling the pulsatile nature of the secretions^21^, the mechanism for the fine control has remained somewhat elusive, mainly due to the GnRH secreting neurons not expressing AR or ER^22^. Investigation suggests that feedback regulation of GnRH neurons is predominantly exerted via kisspeptin cells, found primarily within the anteroventral periventricular nucleus (AVPV) and arcuate nucleus (ARC)—both highly AR (&ER) expressing areas of the brain. Kisspeptin is downregulated by androgens, and by oestrogens derived from testosterone^23^. We could not determine, in our study, if the non-AR staining cells seen in the hypothalamus represented those of the GnRH secreting cells. This indirect feedback system allows integration of both gonadal steroid negative feedback, coupled with signals of ‘reproductive season’—ie daylength, stress, nutritional and immune status, and is thought to couple puberty with growth and nutrition in humans.

The suprachiasmatic nucleus (SCN) was another region of the hypothalamus which showed strong AR (see Fig. 6A). The timing of the circadian rhythm—the master clock, is situated in the SCN, where the day and night light cycles, help it to synchronise. The circadian rhythm organizes a multitude of functions within the body, including the sleep–wake cycle, and hormone secretion. The AR positive SCN neurones receives photic cues from the retina and link day length, time of year to reproductive cycles in animals^24^. Alzheimer’s risk and sleep pattern disruption are strongly correlated, due to SCN deterioration and it may be a confounding factor with lower testosterone in older men. Testosterone and DHT may reduce this effect, indicating a lesser role for ER^25^. Thus, ADT may strongly interfere with the correct neural functioning in response to the coupling of gonadal activity to stress response, nutrition, circadian rhythm and sleep, issues strongly associated with development of neurological disorders.

### Amygdala

The cortical amygdala showed very strong AR nuclear specific staining in a very narrow middle layer of cells (layer 2) see Fig. 6A and showed moderate AR mRNA expression (Fig. 5B), however, this region did not show very strong AR-driven luciferase activity (Fig. 1D).

The amygdala is linked to many diseases/disorders related to behaviour e.g. autism, depression, schizophrenia, and anxiety—which often show sex bias in incidence. In the amygdala, the posterodorsal section, displays neuronal plasticity in response to gonadal hormones. Castration reduces dendritic branching in the posterior medial amygdala and can result in deficient social and sexual behaviour, which could be in part reversed by testosterone treatment. Amygdalar astrocytes default to a “female” pattern with AR dysfunction e.g., testicular feminization syndrome, indicating that androgens and AR are required for cell numbers and neuronal plasticity. Plasticity is seen in adults of both sexes, however, androgens exert more effects in males, indicating a prior embryonic requirement.

The amygdala is linked to two main behaviour types: fear/anxiety and mating behaviours. Chemical signalling molecules (pheromones and odorants) are vital in rodent social communication, determining and controlling reproductive behaviours^21^. Sensory signals identified by the AOB, are processed via the medial amygdala, and transferred to the medial preoptic area, an strongly sexually dimorphic brain region. In humans, uniquely, neuronal links in the amygdala help govern the sense of interpersonal trust^22^. These neuronal pathways antagonized by the testosterone-induced peptide hormone vasopressin. One Testosterone may influence and reduce connectivity between the amygdala and the frontal cortex increasing social vigilance and promoting untrustworthiness, especially concerning facial recognition.

### Pons

AR expression in the hindbrain/pons area showed a highly varied pattern of staining. Throughout the area, patterns of strong nuclear staining was seen in some intermittent cells (15–20% approx.), weak to moderate staining was seen in others, whilst the majority of cells showed no staining (see Fig. 7A, B and D). AR-driven luciferase activity was also modest in this area (Fig. 1D). In situ hybridisation of AR mRNA expression in the mouse accessory olfactory bulb showed a similar pattern of expression (Allen Brain Atlas, ISH Coronal image 35). H&E staining is given in Fig. 7C for reference.

The pons is crucial role in relaying information between parts of the brain, as well as in regulating several essential functions of the body. It serves as a bridge connecting the cerebellum to other parts of the brain and spinal cord. Its functions include coordinating voluntary movements and posture; regulating sleep and wakefulness; controlling the rate and pattern of breathing. Many cranial nerves, which control head and neck functions have their nerve clusters within the pons, and control facial expression, chewing, hearing, and balance. The pons contributes to the regulation of various autonomic functions, including heart rate, blood pressure, and digestion as well as reproductive related behaviour such as avoidance or maternal behaviour.

Interestingly, the pons together with other brain regions have been shown to be integral in the ejaculatory and orgasm response in mammals^21–23^. Therefore, it is not surprising that the fibres leading from the pons strongly express AR. The pons shows embryonic-induced sexual dimorphism in the fibre tracts, and androgens are required to maintain fibre density^24,25^, however, the pons volume itself does not diminish with age^26^, but interference by ADT may lead to impotence or loss of libido^27^.

### Midbrain

Cells in the midbrain area stained very weakly for AR, showing weak nuclear specific staining in only 5–10% of cells (Fig. 7A, B and D), this area showed a modest degree of AR-driven luc activity (Fig. 1D). H&E staining is given in Fig. 7C for reference.

The midbrain is situated between the hindbrain and the forebrain in mammals. It has several important functions related to sensory processing, motor control, and integration of various neural pathways. The midbrain serves as a critical centre for relaying information and coordinating certain behaviours, with roles including sensory processing from visual, auditory, and tactile stimuli and motor control. Degeneration of dopamine-producing cells in this region is linked to Parkinson's disease. It contains the reward and reinforcement centre or ventral tegmental area (VTA), which releases dopamine in response to rewarding stimuli and plays a role in motivation, pleasure, and reinforcement of behaviours and pain processing^21^. Suppression of this area (via ADT) leading to a lack of reward processing, may contribute with anxiety-linked neurological disorders which co-occur with susceptibility to depression.

### Cerebellum

In the cerebellum, the main cerebellar cortex did not show any specific staining for AR, although some degree of background staining. The single cell layer representing the main cell bodies of the Purkinje cells, which lie between the molecular and granular layers (Figs. 8A, B, D and 9A, B, D). AR expression was seen to be very strong in the nuclei, and to some degree, the cytoplasm of the Purkinje cells, although no specific significant AR staining could be seen in the large dendritic bodies of these cells, this could be the background staining seen in the molecular layer where the dendrites lie. AR-driven luciferase activity was not seen in this area (Fig. 1D). In situ hybridisation of AR mRNA expression in the mouse accessory olfactory bulb showed a similar pattern of expression (Allen Brain Atlas, ISH Coronal image 45). H&E staining is given in Figs. 8C and 9C for reference.

The cerebellum controls posture, voluntary movement and vestibular function, and it influences sensorimotor processing. The cerebellum controls and modulates levels of strength and appropriate muscular activation. Although it has long been held that the cerebellum’s functions are purely for motor control, additional roles have been postulated including controlling behaviour and cognition (reviewed in^21,22^).

Purkinje cells are thought to be involved in the fine-tuning of cerebellar circuit motor coordination. The large Purkinje cell dendritic tree-like structures are critical to this process; receiving multiple inputs from the parallel fibres and integrate them into a modulatory signal, which they transfer to the climbing fibres as an error signal that can override or modulate the signal. Over time the motor response becomes refined. Therefore, the Purkinje cell signals can be both depressive and stimulatory depending on the context. This has been previously shown in work using two Purkinje-cell-deprived strains of mutant mice. While this structure is not essential for learning, its absence disturbs the performance and the magnitude of the learned motor response^23^. Purkinje cells respond to circulating testosterone levels in different cerebella areas^24^, and may control or mediate with other brain centres, to control the copulatory behaviour, sexual motivation, and arousal in male mice. Purkinje cells have a very complex hormonal response, being shown to express ER, PR, AR and aromatase at different developmental periods, and their role in the motor responses of copulatory behaviour is still debated. Purkinje cells are also the site of de novo steroid biosynthesis in the brain, mostly neuroprotective progestins. Purkinje cell damage or death can lead to several types of spinocerebellar ataxias and dystonia, and Purkinje cell dendrites are often damaged in certain forms of Alzheimer’s^25^. Again, ADT may disturb physiological responses involved in sexual behaviour, and arousal and may explain the observed loss of libido^26^.

### Medulla

Cells in the medulla stained strongly for AR, with mostly nuclear staining but with some considerable staining present in the cytoplasm (Fig. 9A, B and E). AR staining was present in less frequent in nuclei towards the centre of the medulla. H&E staining is given in Fig. 9C for reference.

The medulla is responsible for autonomic functions and contains the neuronal centres for the cardiac, respiratory, vomiting, and vasomotor control, thus it deals with breathing, heart rate and blood pressure as well as the sleep–wake cycle (reviewed in^21^).

Androgens influence the pons and the medulla, together, to control male sexual behaviour. Genital sensory information is a powerful activator of pre-erectile spinal neurons and produces erections. Some medullar pre-motor neurons, and pons project directly onto spinal sympathetic, parasympathetic, pudendal neurons, and receive sensory information from the genitals^22^. Additionally, they control copulatory behaviour in male mice such as consummatory behaviour^23^. The medulla functions as a hub of sensory integration, and receives inputs from several brain areas, and may regulate several behavioural responses in addition to sexual reproduction, such as pain perception, acoustic startle, breathing and heart rate, aggression, as well as tactile information, and micturition. Many of these behaviours could also be interlinked with sexual behaviour, and sex- specific mating behaviour.

The sexual dimorphism between the sexes regarding autonomic responses has been well studied e.g., in cardiovascular diseases and hypertension, however, the mechanistic evidence is less well understood. Testosterone has effects on the sleep–wake cycle outside its effects via the brain stem. Testosterone replacement in hypogonadal men, increased the number of sleep apnoea’s and hypopneas and wakefulness events^24^.

Kennedy disease (spinal and bulbar muscular atrophy) is categorised by progressive muscle atrophy, weakness, and tremor, where the expression of mutant AR, with expansion of the CAG repeat, can produce a toxic aggregation of the AR protein within neurons of the brainstem and lower motor neurons. The abnormality consists of an X-linked recessive mutation of the AR and mainly affects males^25^.

## Conclusions

The brain’s response to steroid hormones is very complex. For example, even in tissues where AR and ER are both present, it is often unclear as to which receptor is mediating the response to a particular steroid due to T > E_2_ conversion by aromatase and often it is the ER that ultimately conducts the function of the circulating testosterone. Although the main systemic masculinisation effects of androgen are mediated during embryological development, AR is a highly expressed protein within the adult male brain and continues to be an active transcription factor in many different areas. Table 1 summarises the areas in which AR protein is expressed.

Here, we were able to differentiate those regions in which AR itself directly responds to testosterone, by use of the ARE-Luc mouse^23^, which expresses a reporter construct previously validated as not being oestrogen or ER sensitive. The AR-driven luciferase has been shown to be exclusively activated by the ligand-bound AR and only shows luciferase expression in the same cell type as the AR. We observed that brain AR activity was highly specific to small areas—indeed in places to specific layers or clusters of cells—within the brain.

Our data agrees well with a study by Cara et al.^24^, where the main areas of AR immunoreactivity were the stria terminalis, medial preoptic area of the hypothalamus and medial amygdala. All these areas showed strong AR immunoreactivity in our study, differing only in the staining seen for the cerebral cortex piriform area and posterior amygdala which were stronger in our study.

Differences in brain AR activity were evident between the sexes. Overall, brain AR activity was much higher in the male than the female, with the caveat that this is not corrected for either the lower serum circulating testosterone levels within the female or other physiological and morphological differences *e.g.*, sizes of specific brain regions which show dimorphism.

Clinically, outside of prostate cancer treatments, anti-androgens (AR antagonists) are extensively used in males for the treatment of benign prostate hyperplasia, androgenic alopecia, hypersexuality, paraphilias and precociously puberty. In females anti-androgens are used to treat acne, seborrhoea, hidradenitis suppurativa, hirsutism and hyperandrogenism often associated with polycystic ovary syndrome. The physiological effects of both androgens and anti-androgens, outside gonadal tissues, can be very subtle and in some cases diffuse. Additionally in both males and females old age correlates with a decrease in circulating androgens—gradual in the male and much more rapid in post-menopausal women. Therefore, it is relatively unknown as to how much of age-related cognitive disorders may be influenced and indeed rapidly accelerated by loss of androgens.

In hypogonadal men, increasing testosterone levels can significantly help with mood, by increasing serotonin 2A receptor expression in the frontal cortex. However, the more potent but non-aromatisable androgen (DHT) cannot, indicating that this particular event is mediated via conversion of testosterone to oestrogen. Since ER and AR are both required for the correct functioning of the adult male brain, it is interesting therefore that pituitary downregulation ADT, which significantly lowers circulating testosterone, may have more of an effect on the brain, due to diminished T to E2 conversion, whereas androgen antagonists such as bicalutamide may only inhibit the AR-mediated effects in the brain. There is often diversity in ADT modalities, timings and combinations which may increase the complexity in any cognitive studies during ADT.

The levels of circulating androgens and importantly the type of androgen have significant effects on mental health. The 5a reductase inhibitor finasteride has had suicide added to its list of potential side effects^25^, and both low and high testosterone have been implicated in higher rates of suicide in teenage and older men respectively^26^, especially in those with pre-existing conditions such as depression and bipolar disorder^27^.

Genetic mouse models indicate that ER controls prenatal brain masculinization to organize reproductive and territorial behaviours, while postnatal activation of AR strengthens components of those behaviours. These results describe how AR and ER pathways interact to fully masculinize the brain and behaviour of male mice^28^.

The vast majority of the mouse brain did express moderate to high levels of AR RNA and/or protein; this coupled with the extensive AR-driven luciferase activity showed that AR is indeed very active within the male brain. AR was strongly associated with the olfactory system (Olfactory bulb) in the mouse, which is unsurprising given that mouse behaviour and reproduction is strongly governed by odorants and pheromones. Other areas of the brain, including the hypothalamus, cerebral cortex, medulla, and a highly specific cell layer of the cerebellum, showed strong AR protein staining, indicating that androgen and AR are crucially involved in most brain activities and influence or govern many functions of the brain. Although we cannot extrapolate directly to the human brain, nor can we, at his point determine the exact effects of ADT, it supports the supposition that ADT would strongly influence and diminish neural activity and processing in the brain and can lead to impaired cognitive ability, which should not be ignored in the quality-of-life measurements of patients undergoing ADT for PCa therapy.

Although, we have used the mouse here as a model, the mammalian pathways involved are well conserved in humans—responses to pheromones notwithstanding^29^. Pathways involved in odour detection may be directly linked to emotional mood and memory formation, and AR and testosterone have strong effects on our circadian rhythm and daylength discrimination, which are known risk factors for neurological diseases such as Alzheimer’s. Additionally, several centres in the brain involved in copulatory behaviour and arousal are strongly AR responsive and this may explain the fact that loss of libido is a well-documented side effect of ADT. Although the exact effects of ADT are beyond the scope of this study, we hope that this work will serve as a baseline for AR expression and activity in future mouse studies, where the effects of ADT can be scrutinised in more detail, and that the exact AR^+ve^ brain regions, shown here, can be further studied. In conclusion there is a lot we can learn from the mouse ARE-Luc model, least of all that AR is present and transcriptionally active in the adult male mouse brain.

### Caveats

There are some caveats which should also be considered in such a study. Firstly, AR has other effects, outside transcription, and testosterone binding may trigger immediate non-genomic effects leading to membrane depolarisation. Indeed, a membrane associated AR protein, which does not translocate into the nucleus has been postulated^30,31^. Further, strong cytoplasmic AR staining was seen in the glomerular cell layers of the main and accessory olfactory bulbs, without any apparent clear nuclear staining, which was unique to the brain areas studied. The amygdala areas and pons fibre tracts showed both strong cytoplasmic and nuclear staining for AR. This may indicate that other factors may influence AR nuclear import in these cell types.

Secondly, male mice were kept together in groups of 4–5 mice, but in a facility where female mice were also housed in close proximity, therefore, it is unknown whether female mouse pheromones would influence their activity, and also whether close proximity to other males, may cause altered behaviour *e.g.,* increased aggression^32^. Also, male mice kept together may illicit more aggressive tendencies and therefore, we cannot rule out the effects of chronic defeat stress and its impact on testosterone levels and activity^33^, and whether certain males may have altered levels of testosterone as a result.

### Supplementary Information

Supplementary Figure 1.

Supplementary Figure 2.

Supplementary Figure 3.

## Data Availability

Higher resolution larger images of the current study available from the corresponding author on reasonable request. All data generated or analysed during this study are included in this published article.
