# Supplementary material for: Analysis of androgen receptor expression and activity in the mouse brain
Source: Sci Rep. 2024 May 15;14:11115. doi: 10.1038/s41598-024-61733-9 (PMC11096401; doi:10.1038/s41598-024-61733-9)
Supplement: Supplementary file 1 — Supplementary Figure 1. [file 41598_2024_61733_MOESM1_ESM.pdf]

Supplemental Figure 1

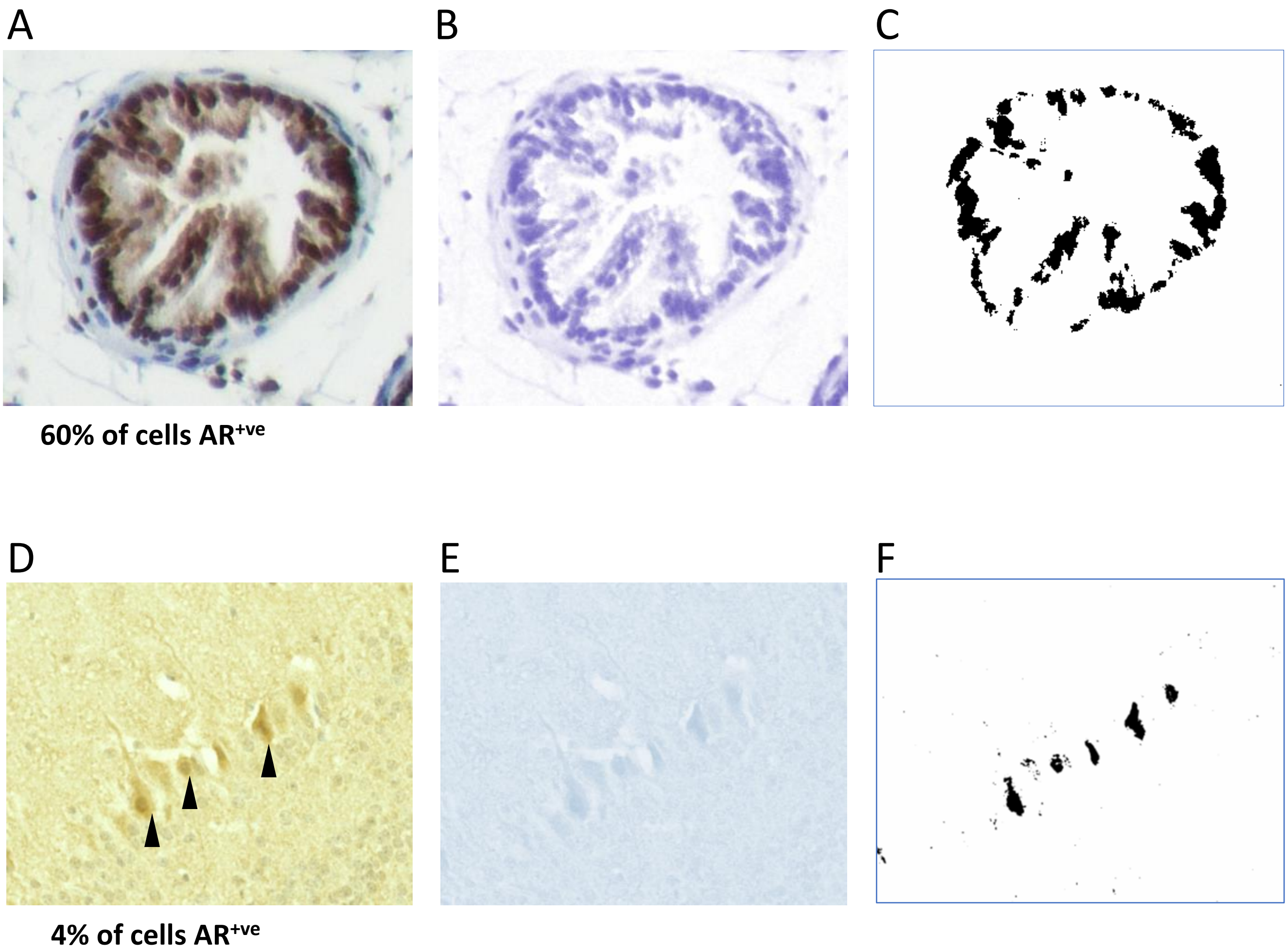

AR Image analysis: **A**, Immunohistochemical staining for AR in normal mouse prostate tissue, showing prostate epithelial cells as a positive control. **B**, colour deconvolution in Image J showing all hematoxylin stained nuclei. **C**, colour deconvolution, detection and binary transformation of diaminobenzidine AR^{+ve} stained nuclei in normal mouse prostate tissue. **D**, Immunohistochemical staining for AR in normal mouse olfactory bulb mitral cell layer. **E**, colour deconvolution in Image J showing all hematoxylin stained nuclei. **F**, colour deconvolution, detection and binary transformation of diaminobenzidine AR^{+ve} stained nuclei in mouse olfactory bulb mitral cell layer. Mitral cells indicated by arrows.
