# Supplementary material for: Analysis of androgen receptor expression and activity in the mouse brain
Source: Sci Rep. 2024 May 15;14:11115. doi: 10.1038/s41598-024-61733-9 (PMC11096401; doi:10.1038/s41598-024-61733-9)
Supplement: Supplementary file 2 — Supplementary Figure 2. [file 41598_2024_61733_MOESM2_ESM.pdf]

Supplemental Figure 2

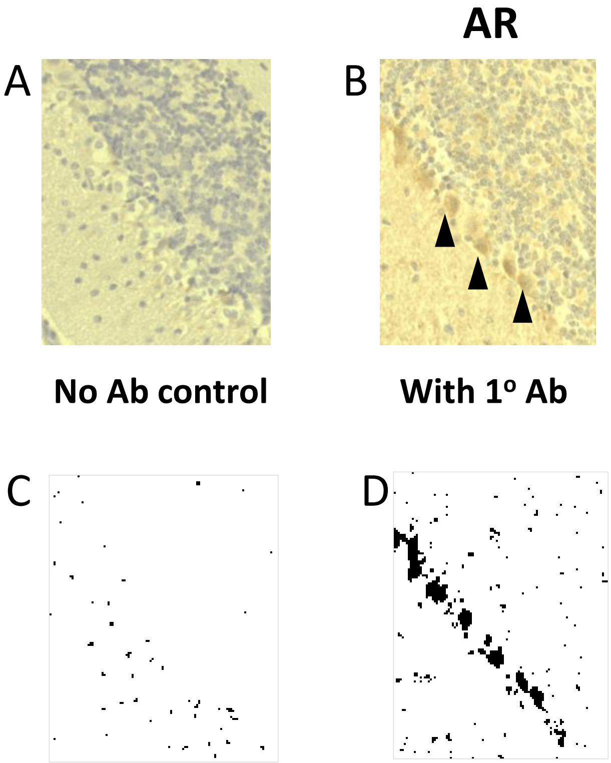

Antibody specificity testing on mouse brain coronal sections of the cerebellum and medulla. **A**, primary antibody omitted. **B**, with primary antibody. **C & D** colour deconvoluted images of diaminobenzidine staining density for AR. Positive AR staining was observed in the Purkinje cell layer of the cerebellum as indicated by the arrows.
