# Supplementary material for: Analysis of androgen receptor expression and activity in the mouse brain
Source: Sci Rep. 2024 May 15;14:11115. doi: 10.1038/s41598-024-61733-9 (PMC11096401; doi:10.1038/s41598-024-61733-9)
Supplement: Supplementary file 3 — Supplementary Figure 3. [file 41598_2024_61733_MOESM3_ESM.pdf]

Supplemental Figure 3

A. Coronal section 1

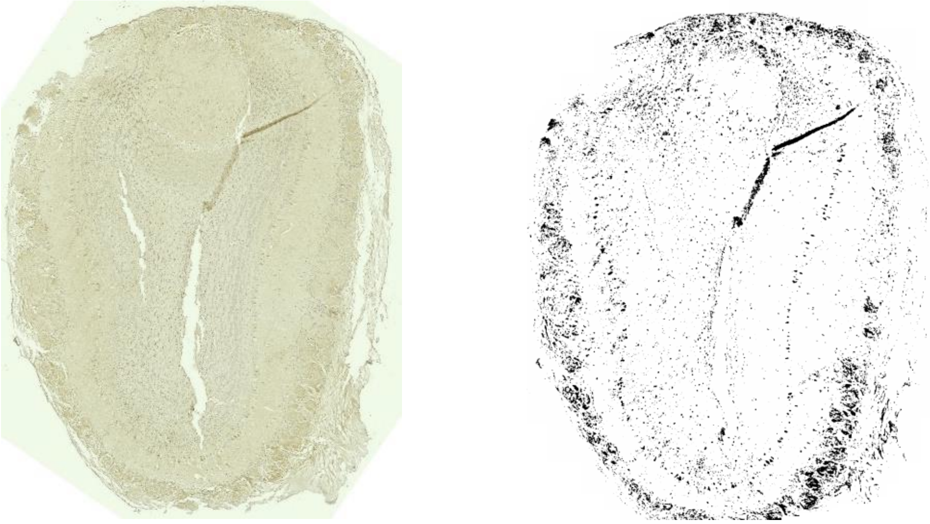

B. Coronal section 2

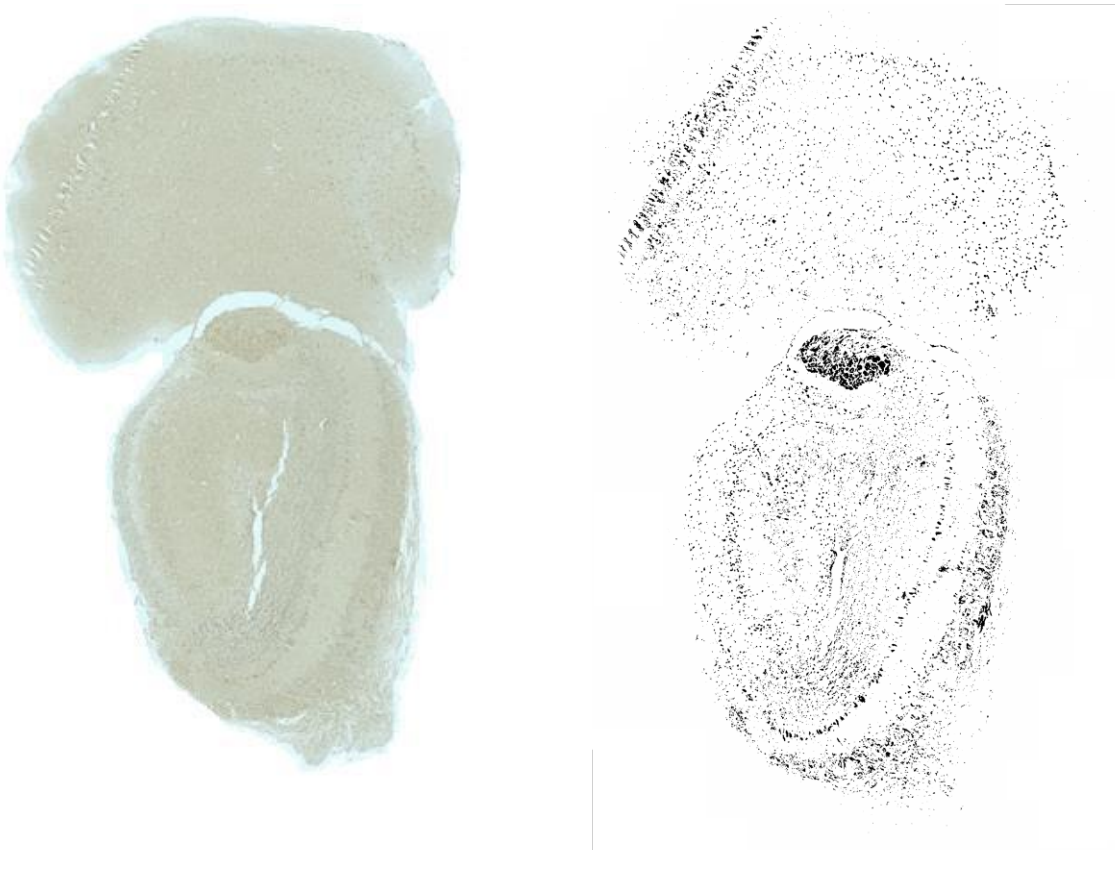

C. Coronal section 3

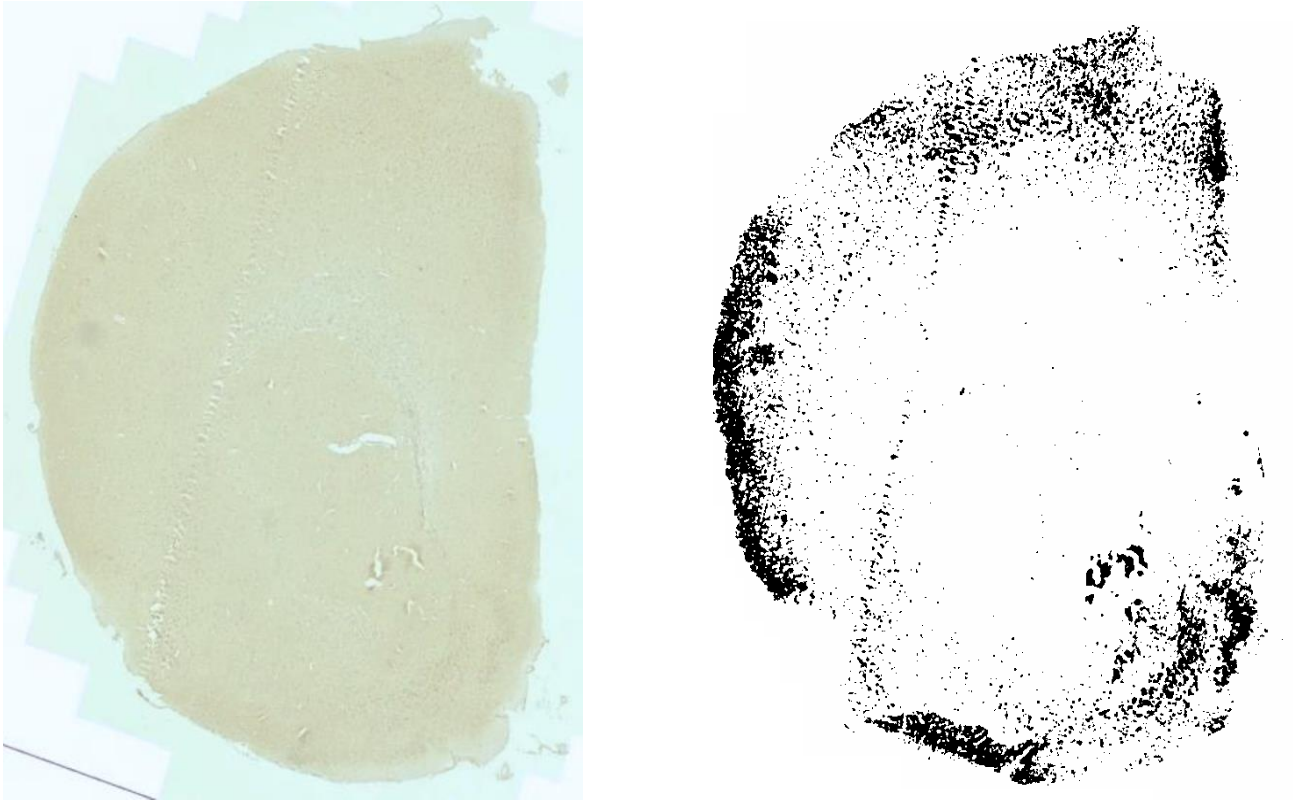

D. Coronal section 4

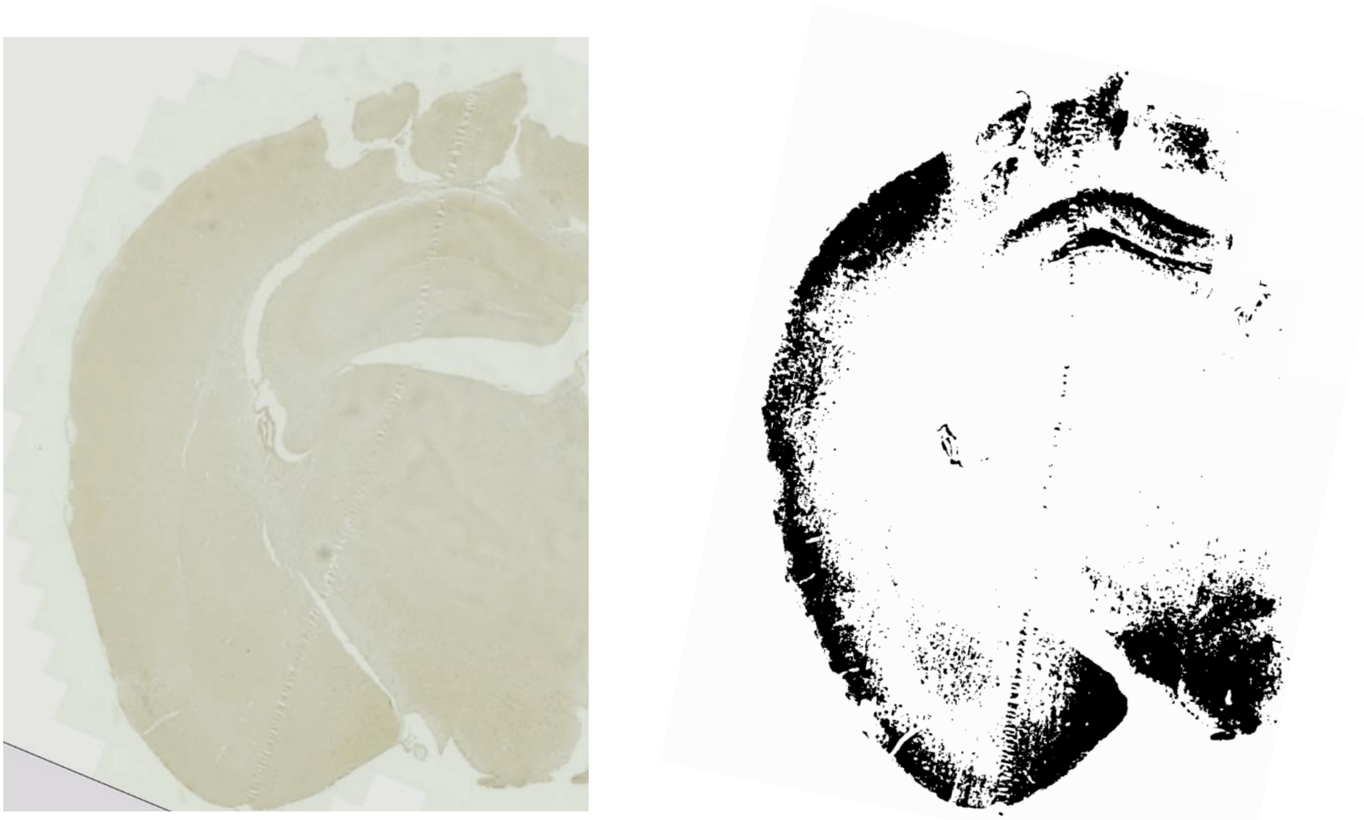

E. Coronal section 5

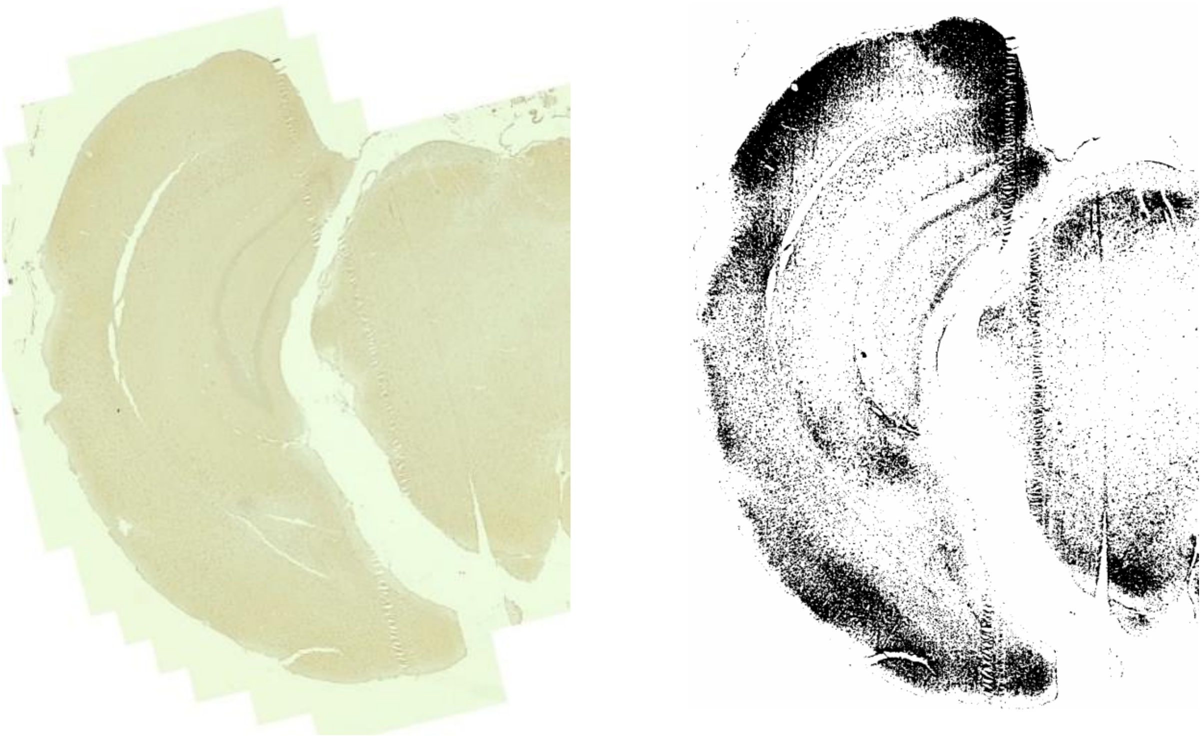

F. Coronal section 6

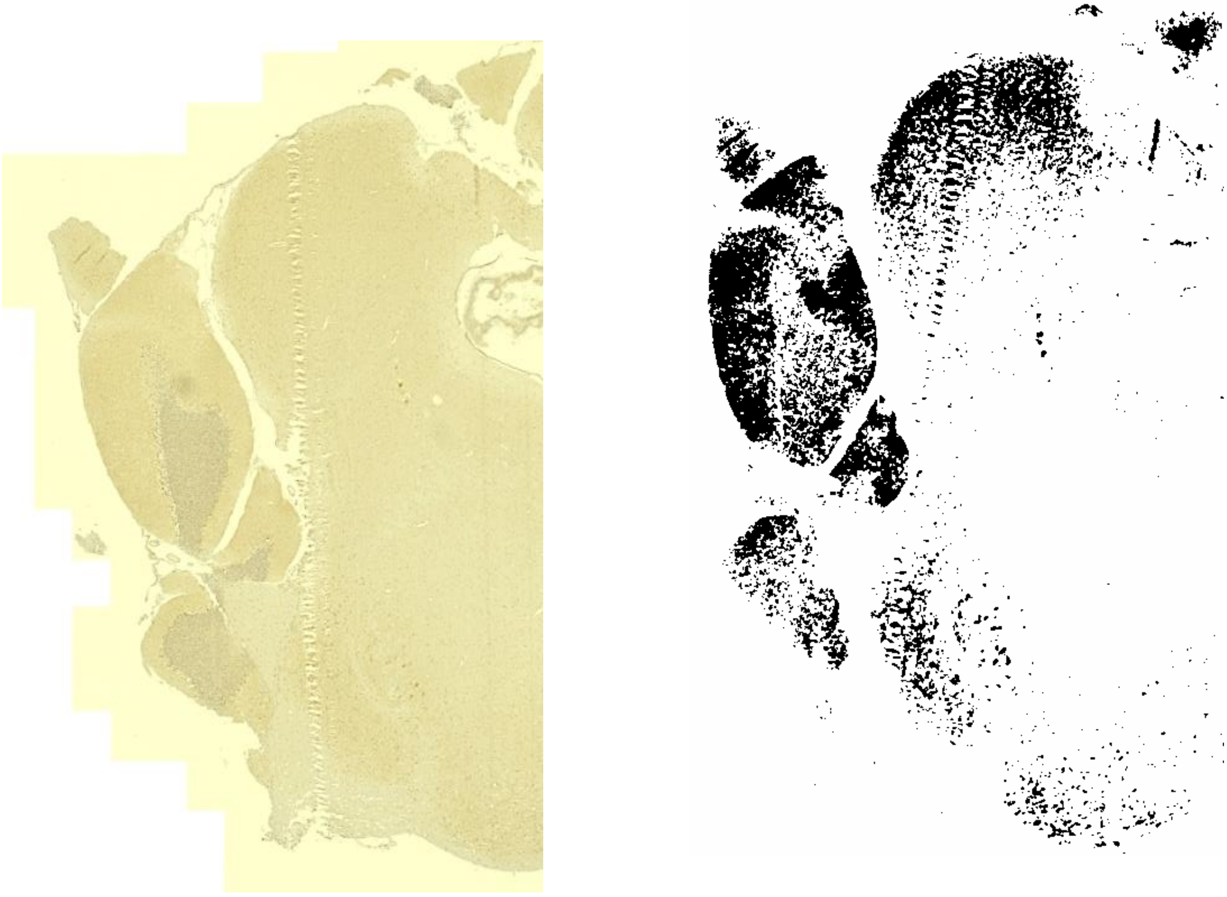

G. Coronal section 8

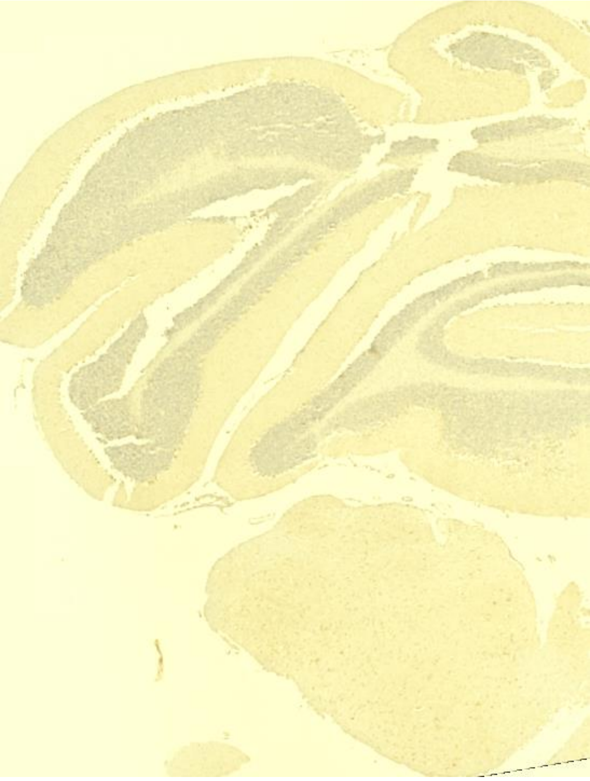

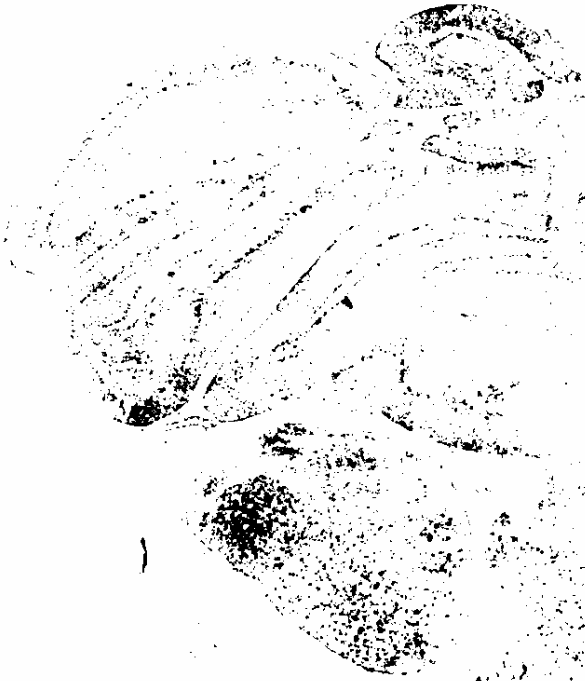

Supplemental Figure 1: Immunohistochemical staining of AR in mouse brain coronal sections (Left hand side), with diaminobenzidine stain colour density (right hand side). Images prepared in Image J.
